# The microRNA in ventricular remodeling: the miR-30 family

**DOI:** 10.1042/BSR20190788

**Published:** 2019-08-02

**Authors:** Xiaonan Zhang, Shaoyang Dong, Qiujin Jia, Ao Zhang, Yanyang Li, Yaping Zhu, Shichao Lv, Junping Zhang

**Affiliations:** 1 Department of Cardiovascular Medicine, First Teaching Hospital of Tianjin University of Traditional Chinese Medicine, Tianjin 300193, China; 2 Department of Orthopaedics, Affiliated Hospital of Hebei College of Traditional Chinese Medicine, Hebei 050011, China; 3 Department of Epidemiology, College of global public health, New York University, 726 broad way, New York, NY 10003, U.S.A.; 4 Department of Integrated Traditional Chinese and Western Medicine, Tianjin Medical University Cancer Institute and Hospital, Tianjin 300193, China

**Keywords:** apoptosis, autophagy, inflammation, miR-30 family, oxidative stress, ventricular remodeling

## Abstract

Ventricular remodeling (VR) is a complex pathological process of cardiomyocyte apoptosis, cardiac hypertrophy, and myocardial fibrosis, which is often caused by various cardiovascular diseases (CVDs) such as hypertension, acute myocardial infarction, heart failure (HF), etc. It is also an independent risk factor for a variety of CVDs, which will eventually to damage the heart function, promote cardiovascular events, and lead to an increase in mortality. MicroRNAs (miRNAs) can participate in a variety of CVDs through post-transcriptional regulation of target gene proteins. Among them, microRNA-30 (miR-30) is one of the most abundant miRNAs in the heart. In recent years, the study found that the miR-30 family can participate in VR through a variety of mechanisms, including autophagy, apoptosis, oxidative stress, and inflammation. VR is commonly found in ischemic heart disease (IHD), hypertensive heart disease (HHD), diabetic cardiomyopathy (DCM), antineoplastic drug cardiotoxicity (CTX), and other CVDs. Therefore, we will review the relevant mechanisms of the miR-30 in VR induced by various diseases.

## Ventricular remodeling

Ventricular remodeling (VR) is a kind of adaptive response to the heart, which is a process of pathological and physiological lesion repair and secondary ventricular systolic dysfunction. It is also an essential process of cardiac hypertrophy and myocardial fibrosis [1]. The number of myocardial cells and myocardial systolic function are decreased in the lesion, and healthy myocardial cells activate the compensation mechanism, resulting in myocardial remodeling to satisfy the stress state of the heart. Hence, the VR maintains the cardiac function in the early stage, but over time, it leads to maladaptive changes, and then the heart function decreased [2]. The VR is found in hypertension, myocardial infarction (MI), and heart failure (HF) [3–5]. It can also be found in diabetic cardiomyopathy (DCM) and the antineoplastic drug-induced cardiotoxicity (CTX) [6,7]. A study has also shown that disease-induced VR is a complex process involving cardiomyocyte growth and death, vascular sparseness, fibrosis, inflammation, and electrophysiological remodeling [8]. In recent years, the medical community has paid more and more attention to the research of VR. It has been recognized that VR is the primary pathological basis for the development of the HF and the determinant of morbidity and mortality [9]. The more severe the VR is, the worse the prognosis is. And severe cases can even cause death.

## MiR-30 family

MicroRNAs (miRNAs) are a class of endogenous, non-coding RNAs with regulatory functions that are about 20–25 nucleotides in length, and are produced by primary polymerases (pri-miRNAs) transcribed by RNA polymerase II (Pol II) [10]. In the nucleus, the pri-miRNA is cut by Drosha and its accessory protein DGCR8/Pasha to generate a pre-miRNA of approximately 70 nucleotides in length [10,11]. The pre-miR is transported from the nucleus to the cytoplasm under the action of the transporter Exportin-5 and is cut into a double-stranded miRNA of about 20–25 nucleotides under the action of Dicer enzyme [10]. The double-stranded miRNA is then loaded into a RNA-induced silencing complex (RISC) in which one strand is degraded, and the other strand remains of the complex to form a mature miRNA [10,11]. The miRNA binds to the 3′ UTR of the target gene mRNA by specific base pairing, induces degradation of the target mRNA or inhibits its translation into a protein [12]. A miRNA can act on multiple target mRNAs, and multiple miRNAs can also work as a functional cluster to coordinate the expression of a particular gene. It is involved in cell proliferation, differentiation, apoptosis, and angiogenesis, etc. [13]. And the miRNA plays a vital role in the pathophysiology of cardiovascular diseases (CVDs) such as myocardial infarction, hypertrophy, fibrosis, HF, etc. [14].

The miR-30 family is abundantly expressed in the heart, and its expression is significantly decreased after cardiomyocyte hypertrophy and myocardial ischemia/reperfusion [15,16]. And another study has also shown that miR-30 is involved in VR [17]. The miR-30 family is composed of miR-30a, miR-30b, miR-30c, miR-30d, and miR-30e, while miR-30c is further divided into miR-30c-1 and miR-30c-2. The miR-30 family is encoded by six genes located on human chromosomes 1, 6, and 8 [10,13]. These miRNAs share the same seed sequence, but have different compensatory sequences near the 3′ end, allowing the miR-30 family members to target different genes and pathways to perform diverse biological functions [13].

## Mechanism of action of the miR-30 family

At present, the research on miR-30 family has been gradually carried out, and its biological function has been initially clarified. The miR-30 family is involved in cell growth and tissue differentiation, which is also closely related to the disease [18]. There is increasing evidence that miR-30 has potential roles in a variety of physiological and pathological states. When the expression level of the miR-30 family is dysregulated, it mediates the corresponding mechanism by regulating different target genes. And a series of studies have shown that the miR-30 family plays a varying degree of regulation on autophagy, apoptosis, oxidative stress, and inflammation [19–24], the related research is as follows.

### Autophagy

The miR-30 family has been shown as an autophagy inhibitor in some biological processes [25]. MiR-30 targets Beclin1 mRNA, which mediates autophagy. In the experimental model of cardiac hypertrophy rats, miR-30a expression is down-regulated, Beclin1 expression is up-regulated, and cardiomyocyte autophagy is enhanced [26]. Also, in medulloblastoma, miR-30a inhibits autophagy by down-regulating the expression of Beclin1 [27]. *In vitro* and *in vivo* experiments of doxorubicin-induced cardiomyopathy, miR-30e was also found to down-regulate the expression of Beclin1 [28]. The antineoplastic drug 5-FU may inhibit miR-30 to up-regulate Beclin-1 to induce autophagic cell death and cell proliferation arrest [29]. Endothelial cell injury is an essential mechanism for the development of atherosclerosis. Atherosclerosis is a common risk factor of the CVDs. The levels of autophagy-associated protein 6 (ATG6) and miR-30 in atherosclerotic ApoE (-/-) mice were measured by high-fat diet (HFD) or healthy diet, and the potential relationship between the two was predicted. It was found that HFD-induced miR-30 increase, targeted inhibition ATG6 mRNA translation results in the decrease of endothelial cell autophagy level significantly, endothelial function is damaged [25]. It has also been found that overexpression of miR-30d significantly inhibits the proliferation of colon cancer cells by directly targeting mRNA of ATG5, phosphoinositide 3-kinase, and Beclin1, and is involved in promoting apoptosis and autophagy inhibition [19]. In conclusion, miR-30 targeting Beclin1 or ATG mRNA regulates the level of autophagy.

### Apoptosis

The miR-30 family not only regulates autophagy but also involves apoptosis. The miR-30 may participate in different pathways by targeting different genes [30]. The related research has shown miR-30 has an anti-apoptotic effect, and its inhibitors can reduce mitochondrial oxygen consumption, release Cytochrome C, and activate caspase 3 and 9, thereby activating the mitochondrial apoptotic pathway [31]. MiR-30a can specifically inhibit the Snail mRNA expression associated with non-small-cell lung cancer (NSCLC). In NSCLC tissues, miR-30a expression was significantly down-regulated, while Snail expression was up-regulated considerably, which can decrease E-cadherin and occludin expression, and promote N-cadherin and vimentin expression to induce apoptosis [32]. MiR-30a overexpression can also inhibit MEF2D and GRP78, reduce apoptosis, and promote the growth of lung cancer and cell renal cell carcinoma (ccRCC) cells, respectively [33,34]. Besides, miR-30c was involved in the regulation of cisplatin-induced apoptosis in renal tubular cells by targeting the BNIP3L and Hspa5 [35]. MiR-30e can directly target BCL2/adenovirus E1B interacting protein 3 (BNIP3L) gene, and its overexpression targeting BNIP3L attenuates aldosterone-induced podocyte apoptosis and mitochondrial dysfunction, whereas silencing miR-30 leads podocyte apoptosis and mitochondrial dysfunction [36]. In diabetic nephropathy (DN), miR-30 inhibitors result in increased expression of the oncogene Mtdh, which activates the p38 MAPK-dependent pathway to promote podocyte apoptosis [21]. Moreover, transforming growth factor β 1 (TGF-β), a key mediator of podocyte injury, induces down-regulation of miR-30 to promote apoptosis [37].

### Oxidative stress and inflammation

Oxidative stress and inflammation also play regulatory roles in microRNA signaling in CVDs [38]. Oxidative stress may be an essential factor in miRNA expression in some cells, such as human retinal pigment epithelial cell line (ARPE-19) [23,39]. MiR-30b-5p may be associated with oxidative stress and up-regulated with increasing H_2_O_2_ concentration [23]. The research has also shown that miR-30 is dysregulated in the atherosclerosis of the neck and lower limbs, which is involved in the regulation of angiogenesis, endothelial cell function, inflammation, cholesterol metabolism, oxidative stress, and extracellular matrix composition [40]. And in spinal cord injury (SCI), miR-30a-5p targeting Neurod 1 through MAPK/ERK signaling has been shown to improve inflammatory response and oxidative stress [41]. MiR-30a-5p and miR-30d-5p can regulate inflammation through indirect or direct mechanisms, which are negatively correlated with pro-inflammatory cytokine levels and thus negatively regulates inflammation [42]. Similarly, the expression of miR-30a-5p, miR-30c-5p, and miR-30e-5p was down-regulated in macrophages (ATM) of HFD mice, which mediates Notch1 signaling to drive chronic low-grade inflammation [24]. In addition, one study has shown that lincRNA-p21 enhances TGF-β signaling through interaction with miR-30, mediating liver fibrosis, and inflammation. And the present study has also demonstrated that ectopic expression of miR-30 in hepatocytes reduces CCI4-induced hepatic fibrosis and inflammation [43]. Therefore, the miR-30 family is closely related to oxidative stress and inflammation.

## Ventricular remodeling and MiR-30 family

VR is a risk factor for adverse cardiac events and is closely related to sudden cardiac death, arrhythmia, and MI [44]. It is mainly involved in fibroblasts increasing, collagen fiber deposition, myocardial apoptosis, and myocardial cell hypertrophy, etc., which are essential causes of myocardial dysfunction, HF, and even death [45]. In recent years, more and more experimental data have confirmed the importance of miRNAs in CVDs. Some miRNAs have also been particularly closely watched because they are involved in regulating cardiac-related function and have an impact on VR [46]. Studies have also shown that oxidative stress, inflammation, autophagy, apoptosis, and other cellular mechanisms are involved in the remodeling of the heart [47–49]. The miR-30 family participates in autophagy, apoptosis, oxidative stress, and inflammation through post-transcriptional regulation of target gene proteins, directly or indirectly involved in various disease including ischemic heart disease (IHD), hypertensive heart disease (HHD), DCM, antineoplastic drug CTX, and other disease-induced VR. Therefore, the relevant studies on VR induced by multiple diseases and miR-30 family are summarized as follows.

### Ischemic heart disease

IHD is mainly caused by coronary artery occlusion and is the leading cause of death worldwide [50]. It is a myocardial ischemic injury caused by the imbalance of coronary blood flow and myocardial demand. Once the myocardium is in a long-term ischemic state, it will lead to the necrosis and loss of myocardial cells, further lead to VR, and eventually cause HF [51]. The mechanism of IHD mainly includes energy metabolism disorder, increased oxygen free radical production, calcium overload, endothelial dysfunction, inflammatory reaction, and mitochondrial damage, but the specific molecular mechanism is still unclear [52–56].

The study found that miR-30 family expression disorder is closely related to IHD. The expression of miR-30 was significantly decreased, leading to a decrease in the ability to down-regulate XBP1. XBP1 regulates the autophagy signaling pathway through Beclin-1 transcription, and sustained activation of XBP1 leads to endothelial cell apoptosis and promotes the development of atherosclerosis [57,58]. In animal models of cardiac hypertrophy and patients with HF, the expression of miR-30 is decreased, while the expression of XBP1 and its downstream target gene vascular endothelial growth factor (VEGF) is increased, promoting angiogenesis [57]. The miR-30 family also targets DLL4 and indirectly regulates VEGFA signaling, which plays a vital role in vascular development and angiogenesis [59]. Besides, in the process of the HF, the expression of miR-30 is decreased, and the expression of GALNT1 and 2 is up-regulated, which in turn increases the proportion of proBNP secreted by the heart and impairs the compensatory effect of circulating BNP [60]. The miR-30-5p showed vigorous activity throughout MI and was closely related to apoptosis. Under hypoxic conditions, miR-30-5p directly acts on the target apoptosis genes Picalm and Skil, protecting cardiomyocytes from apoptosis and necrosis [61]. Another related study showed that silencing miR-30e inhibited significantly the expression of the apoptosis regulators Bax and caspase 3 in H9C2 cells, thereby inhibiting apoptosis [62]. This conclusion is contrary to the anti-apoptotic mechanism of miR-30, which needs further research to confirm.

MiR-30 also significantly increased p62 and Beclin-1 expression, activated autophagy, and acted on the Notch1/Hes1/Akt signaling pathway, reducing inducible nitric oxide synthase (iNOS) and participating in oxidative stress protein expression [62]. Another study has shown that miR-30 may regulate the proinflammatory polarization of adipose tissue macrophages via DLL4-mediated Notch1 signaling, which constitutes an inflammatory response to cardiac metabolic disorders [24]. In addition, Wang et al. showed that the long non-coding RNA (lncRNA) n379519 binds directly to miR-30 and exhibits a negative regulatory trend [63]. In MI mice, the expression of lncRNA n379519 was significantly up-regulated, promoting collagen deposition and cardiac fibrosis. However, there is still a study showed that the expression of the miR-30 family was up-regulated in the mouse MI model and the primary cardiomyocyte hypoxia model, while the expression of cystathionine-γ-lyase (CSE) was significantly reduced, and the production of cardioprotective hydrogen sulfide (H_2_S) was decreased, thereby aggravating the damage of hypoxic cardiomyocytes [64,65]. In contrast, silencing the miR-30 family can prevent hypoxic cell damage by increasing CSE and H_2_S levels [65]. The expression level of miR-30 in this MI conflicts with the previous result. In conclusion, these studies have shown that miR-30 is involved in the development of the IHD, and its expression level and regulation mechanism still need to be further verified by a large number of experiments.

### Hypertensive heart disease

HHD is a heart disease caused by arterial hypertension, which can also cause HF, IHD, and left ventricular hypertrophy (LVH) [66]. LVH is a crucial link in the process of hypertension, characterized by inappropriate myocardial fibrosis, and LVH and myocardial fibrosis are associated with poor prognosis [67]. LVH is also an early stage of the cardiovascular event chain and is an independent risk factor for morbidity and mortality such as coronary heart disease (CHD), HF, arrhythmia, and sudden cardiac death [68]. The formation and development of HHD pathological process are closely related to the renin-angiotensin system (RAS), endothelial cell function, inflammatory response, and the like [69–72].

Although there are relatively few studies on miR-30 family and HHD, it is still found that the miR-30 family directly or indirectly affects the pathogenesis of HHD. In the RAS system, angiotensin II (Ang II) is the most critical effector molecule [69]. Ang II induces cardiomyocyte hypertrophy, heart damage, and fibrosis [73]. Dang et al.’s [74] data suggest that Ang II also causes oxidative stress, inflammation, and mitochondrial damage, which leads to apoptosis and endothelial senescence. The binding of Ang II to the AT1 receptor can cause apoptosis of cardiomyocytes, resulting in a series of complex reaction processes such as decreased Bcl-2/Bax and caspase-3 activation [75]. Ang II, mediated by AT2 receptor, also induces apoptosis of ventricular endothelial cells, which affects the function of endothelial cells as a physical barrier between blood and myocardial cells, thus affecting the function of myocardial cells [70]. When endothelial cells are disturbed by blood flow, the miR-30 family members are up-regulated by shear stress and the flow-induced transcription factor KLF2. And overexpression of the miR-30-5p family members targets the inhibition of Ang II and reduces the expression of inflammatory cell adhesion molecules [71]. Hypertension is also a multifactorial chronic inflammatory disease that leads to cardiac remodeling. A-kinase ankyrin 12 (AKAP12)-/- promotes Ang II-induced oxidative stress, transforming growth factor β 1 (TGF-β1)-mediated inflammation, apoptosis, autophagy, and fibrosis, which also affects cardiac function [72].

Circulating miR-30 may be an essential marker for the diagnosis of HHD. The overexpression of miR-30c reduces the level of connective tissue growth factor (CTGF), a key molecule in the process of fibrosis, and the reduction of collagen production accompanies the decrease of its level. Decreased miR-30 in pathological LVH leads to increased CTGF levels, which contributes to collagen synthesis [17]. Pan et al. [26] found that Ang II-induced down-regulation of miR-30 in cardiomyocytes mediates overexpression of the Beclin-1, which in turn promoted cardiac hypertrophy through excessive autophagy. With the increase of myocardial fibrosis, the expression of myocardial miR-30a decreased, which leads to a significant increase in Snail1 expression, suggesting that miR-30a might be involved in the pathogenesis of myocardial fibrosis through Snail1 [76]. Another study has shown that hypertensive HF is closely related to calcium/calmodulin, which is involved in the cardiac remodeling process [77]. The miR-30 regulates calcium/calcineurin signaling by targeting key factors such as TRPC6, PPP3CA, PPP3CB, PPP3R1, and nuclear factor of activated T cell 3 (NFATC3). This process is triggered by the movement of Ca^2+^ from the extracellular space into the cell, and Ca^2+^ binds to calcineurin and activates its phosphatase activity. Activated calcineurin dephosphorylates NFATC family members and transfers to the nucleus as a transcription factor to regulate gene expression, which plays an essential role in cardiac hypertrophy [78].

### Diabetic cardiomyopathy

DCM is one of the significant complications of diabetes. It is characterized by changes in myocardial structure and cardiac function caused by diabetes, including ventricular hypertrophy, myocardial fibrosis, and decreased cardiac function, and even HF [79]. Insulin resistance, hyperinsulinemia, and hyperglycemia are independent risk factors for the development of DCM [80]. At present, VR is also considered to be a crucial link in the development of DCM and is the most critical factor affecting the prognosis of patients. It can cause cardiac systolic and diastolic dysfunction, decreased ejection fraction, microvascular disease, interstitial inflammation, oxidative stress, and intracellular calcium imbalance [81]. The pathogenesis of DCM is complex, which may involve various factors such as cardiac hypertrophy, myocardial fibrosis, cardiomyocyte apoptosis, and myocardial metabolic disorders, which has a significant impact on the occurrence and development of DCM [82].

The miR-30 family is the basis for regulatory signaling events involved in pancreatic epithelial cell responses during mesenchymal transformation, which is closely related to diabetes [83]. At the same time, miR-30c is anti-hypertrophic, targeting the critical regulators of cardiac hypertrophy and apoptosis, p53 and p21, but the miR-30c expression was down-regulated in diabetic-induced cardiac hypertrophy and fibrosis [84]. Another study also showed that miR-30c was significantly decreased, and the expression of target p53 was increased, which mediated the activation of cell signal transduction pathway, leading to cardiomyocyte hypertrophy and apoptosis [85]. Besides, miR-30c was significantly down-regulated in the myocardial cell of DCM, and Cdc42 and Pak1 target gene expression were increased, mediating pro-hypertrophic effects of hyperglycemia [86]. Li et al. [87] found that streptozotocin (STZ) induced diabetic rats and high glucose-treated cardiomyocytes have significantly increased miR-30d expression and directly inhibited foxo3a expression and its downstream proteins, which in turn up-regulate caspase-1 and the pro-inflammatory cytokines IL-1β and IL-18, promote apoptosis and programmed cell death of inflammatory cells. It suggests that miR-30d may be a potential target for the treatment of DCM. In the pathogenesis of Type 2 diabetes (T2DM), a classic feature is the loss of function of islet β cells, and islet β cell apoptosis is associated with the activated JNK pathway. MiR-30d-mediated direct inhibition of SOCS3 acts to protect islet β cell function through the JNK signaling pathway [88].

In summary, miR-30d can regulate both foxo3a and SOCS3, and play different roles by regulating different mRNAs. In a randomized trial of diabetic mice, miR-30a mediates autophagy, but the specific association with the two has not been explored [89]. In DCM, autophagy is closely related to oxidative stress, which can accelerate the decomposition of free fatty acids and glycosylation products, reduce the damage of harmful metabolites to cells, improve the hypoxia and metabolic disorder of diabetic myocardium, and thus reduce the occurrence of oxidative stress [90,91].

### Antineoplastic drug CTX

The CTX is one of the most common adverse reactions of antineoplastic drugs in the treatment of tumor diseases. The CTX, usually caused by antineoplastic medications, can be divided into type I and type II, mainly manifested as myocardial cell damage. In particular, type I CTX caused by anthracyclines (ANTs) and traditional chemotherapeutic drugs, usually shows as irreversible myocardial cell damage [92]. Clinically, the CTX caused by ANTs can be seen in the left ventricular wall thickening, accompanied by diastolic dysfunction, HF and other manifestations [7], and a large body of evidence suggests that oxidative stress, inflammation, myocardial mitochondrial dysfunction, and apoptosis are involved in the CTX [93–95]. However, the molecular mechanism of action of the antineoplastic drug CTX has not yet been fully clarified.

The miR-30 family was closely related to tumor and CVDs. The study found that the miR-30 family induced myocardial fibrosis and was involved in autophagy and apoptosis of tumor cells [76,96]. There was a study that showed that doxorubicin could induce obvious CTX in mice, such as cardiac dysfunction, through activating Akt-mediated cardiomyocyte excessive autophagy and apoptosis, suggesting that autophagy and apoptosis were also involved in the CTX of antineoplastic drugs [97,98]. Among them, miR-30e was involved in doxorubicin-induced cardiac dysfunction, and the study showed that angiotensin-converting enzyme 2 (ACE2) retains autophagy in cardiomyocytes via the miR-30e/Beclin-1 autophagy signaling pathway, thereby attenuating doxorubicin-induced cardiac dysfunction [28]. In another study of doxorubicin-induced cardiomyocyte injury, it was found that doxorubicin caused down-regulation of the miR-30 family through GATA-6, while overexpression of miR-30 protected cardiomyocytes from doxorubicin-induced apoptosis [99]. Similarly, the study has also shown that overexpression of miR-30a can significantly promote chemotherapy-induced apoptosis and reduce autophagy activity responding to chemotherapy [96]. Hence, the miR-30 family is closely related to antineoplastic drug CTX.

The polycomb group protein enhancer of zeste homolog 2 (EZH2) is an essential regulator of various malignancies. Up-regulation of EZH2 in malignant peripheral schwannomas (MPNSTs) inhibits transcription of miR-30d by the promoter of binding activity. It leads to enhancing the expression of the nuclear transporter receptor KPNB1 and promotes tumor development [100]. The doxorubicin also mediates inflammatory responses by increasing levels of pro-inflammatory cytokines such as IL-1β and nuclear factor kappa-B (NF-κB), which are involved in doxorubicin-induced CTX [101]. Moreover, overexpression of NF-κB increased the expression of miR-30a-5p, inhibited the expression of its target gene WWP1, and promoted the malignant behavior of glioma cells. In contrast, WWP1 overexpression reduced miR-30a-5p expression by inhibiting NF-κB, thereby inhibiting the vicious behavior of glioma cells [102]. It can be seen that miR-30 is closely related to inflammation in antineoplastic drug CTX. In summary, the miR-30 family is associated with apoptosis, autophagy, and inflammation in the antineoplastic drug CTX, but its specific relationship with antineoplastic drug-induced VR also needs further research.

### Other related diseases

VR can also be seen in diseases such as dilated cardiomyopathy, viral myocarditis (VMC), and pulmonary hypertension (PH) [2,103,104]. Dilated cardiomyopathy is characterized by left ventricular, right ventricle, or bilateral ventricular enlargement, with myocardial fibrosis, cardiac hypertrophy, and myocardial systolic dysfunction in the absence of abnormal load conditions and severe coronary artery disease [103,105]. Although many studies have been devoted to exploring the pathogenesis of dilated cardiomyopathy, it is still unknown to a large extent. Extracellular matrix (ECM) fibrosis is one of the main characteristics of dilated cardiomyopathy, and miR-30 is involved in the process of ECM fibrosis by regulating TGF-β1 and CTGF protein levels [106,107]. The study has shown that miR-30c and miR-30d are down-regulated in a model of dilated cardiomyopathy of mice, which mediates apoptosis signal effectors and leads to loss of cardiomyocytes, HF, and dilated cardiomyopathy [108]. VMC, whose myocardial manifestations are inflammatory reactions, and ventricular fibrin deposition increases, the heart continues to remodel, and eventually, the development of dilated cardiomyopathy and HF [2]. MicroRNAs are involved in the pathogenesis of VMC, such as miR-1, miR-146b, and miR-21 [109,110]. MiR-21 may promote myocardial fibrosis in VMC mice via the TGF-β1/Smad7 protein signaling pathway [109]. The microRNA can be used to predict and diagnose VMC, but the direct link between VMC and miR-30 family has not been reported yet. Therefore, we need to study the relationship between the two in VMC further. PH is characterized by a gradual occlusion of the pulmonary (micro) vasculature, resulting in increased vascular resistance, which in turn causes right ventricular hypertrophy and failure [104]. Liu et al. [111] showed that TGF-β1 in PH could increase the expression of Bcl2 by activating the PI3K/Akt signaling pathway, thereby inhibiting the apoptosis of pulmonary artery smooth muscle cells and inducing pulmonary vascular remodeling. In addition, TGF-β1 can target different target genes to play different regulatory roles, and in the heart, it mediates Smad3 signaling involved in myocardial fibrosis [112]. In another chronic hypoxia or monocrotaline-induced PH study, TGF-β1 signaling may mediate down-regulation of miR-30 [113]. These results also suggest that TGF-β1, miR-30 has a potential link with PH-induced ventricular remodeling, but its specific exact relationship needs further study.

## Conclusion

**Figure 1:**
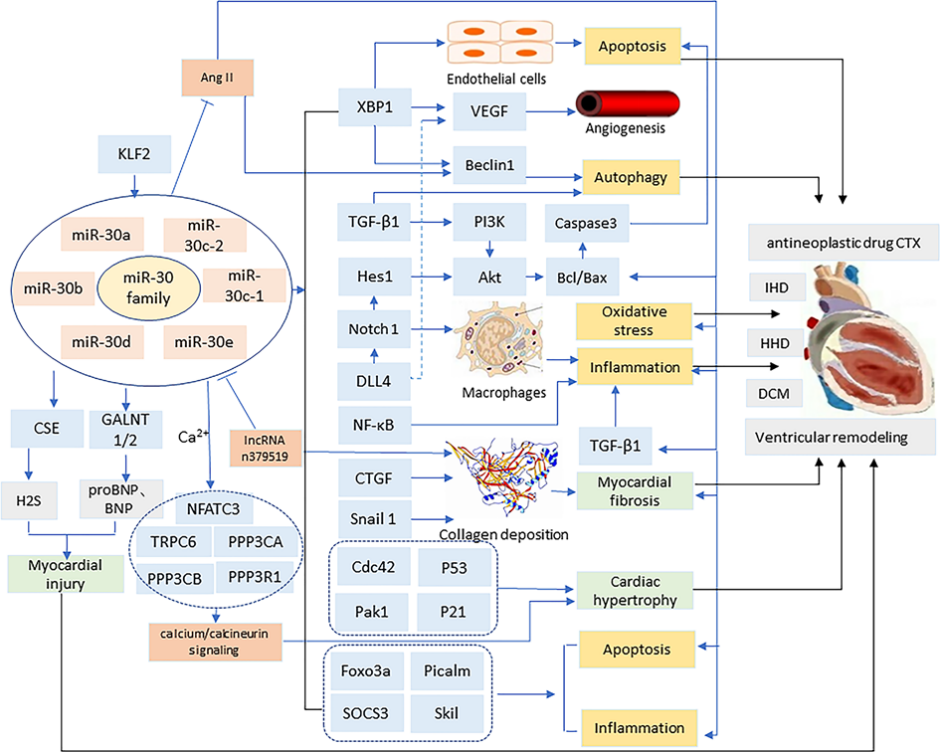
The possible mechanism of miR-30 family mediating ventricular remodeling

In summary, the miR-30 family can regulate autophagy, apoptosis, oxidative stress, and inflammation by targeting XBP1, TGF-β1, CTGF, and other mRNAs, which are involved in the pathogenesis of VR associated with different diseases such as IHD, HHD, DCM, antineoplastic drug CTX, and other CVDs (Figure 1). However, the conclusions of the miR-30 family on apoptosis are still controversial. They may be related to differences in regulatory mRNAs or the detection bias. In short, the results in this area need further analysis and verification. Generally, overexpression of miR-30 family can inhibit autophagy, anti-apoptosis, reduce inflammation, etc., thereby reducing collagen deposition, myocardial fibrosis, and prevention of cardiac hypertrophy. There are few studies on the exact mechanism of action of the miR-30 family on VR at present. The existing research is not deep enough, and more research is needed to verify these conclusions. Thus, in the future, it is necessary to conduct extensive and in-depth research on the potential links between VR and miR-30 family to prevent and treat VR.

## Abbreviations

AKT: protein kinase B
ANG II: angiotensin II
ANT: anthracycline
ATM: macrophages
BNIP3L: BCL2/adenovirus E1B interacting protein 3
CSE: cystathionine-γ-lyase
CTGF: connective tissue growth factor
CTX: cardiotoxicity
CVD: cardiovascular disease
DCM: diabetic cardiomyopathy
DLL4: delta-like 4
ECM: extracellular matrix
EZH2: enhancer of zeste homolog 2
H_2_S: hydrogen sulfide
HF: heart failure
HFD: high-fat diet
HHD: hypertensive heart disease
IHD: ischemic heart disease
lncRNA: long non-coding RNA
LVH: left ventricular hypertrophy
MI: myocardial infarction
miRNA: microRNA
miR-30: microRNA-30
NF-κB: nuclear factor kappa-B
NFATC: nuclear factor of activated T cell
NOTCH1: notch homolog protein–1
NSCLC: non-small-cell lung cancer
PH: pulmonary hypertension
RAS: renin–angiotensin system
SOCS3: suppressor of cytokine signaling 3
TGF-β: transforming growth factor β
VEGF: vascular endothelial growth factor
VMC: viral myocarditis
VR: ventricular remodeling
